# Population Dynamics and Conservation Implications of the Naturally Rewilded Milu Deer in Dongting Lake

**DOI:** 10.3390/ani16213519

**Published:** 2026-11-06

**Authors:** Yunyun Zhou, Fen Xiao, Yucheng Song, Dali Gao, Xiaoqin Liu, Xinman Gui, Fen Song, Shurong Tian, Xiaojie Gui

**Affiliations:** 1 Hunan Academy of Forestry, Changsha 410004, China; zyyswdyx@hnlky.cn (Y.Z.); songfen7777777@163.com (F.S.); tsrswdyx@hnlky.cn (S.T.); 2 Yuelushan Laboratory, Changsha 410004, China; 3 State Key Laboratory of Woody Oil Resources Utilization, Changsha 410004, China; 4 Administration of East Dongting Lake National Nature Reserve, Yueyang 414017, China; sycheng1113@163.com (Y.S.); gaodali0113@163.com (D.G.); 5 Forestry Department of Hunan Province, Changsha 410004, China; liuxiaoqin2026@163.com; 6 Dalhousie University, Halifax, NS B3H 4R2, Canada; qi673140@dal.ca

**Keywords:** population dynamics, population growth model, *Elaphurus davidianus*, endangered species, reintroduction, Dongting Lake

## Abstract

Population dynamic analysis provides a scientific basis for endangered species conservation strategies. The Milu deer (*Elaphurus davidianus*), endemic to China and once classified as Extinct in the Wild by the IUCN, has become one of the world’s most successful endangered species reintroduction cases since 1985. After naturally dispersing to Dongting Lake in 1998, the species established the world’s only naturally rewilded population, which was further expanded and genetically optimized via artificial population supplementation. Focusing on this naturally rewilded population, this study used 20 years of synchronous survey data (2006–2025) to estimate model parameters via Bayesian inference and nonlinear least squares (NLS). We developed exponential and logistic growth models, analyzed the population’s dynamic characteristics and their influencing factors, simulated growth trends under scenarios with and without population supplementation, and explored the relationships between hydrological factors and population size. The results showed that the population increased from 27 to 400 individuals with an average annual growth rate of 0.16 ± 0.03, with the fitted models indicating that rapid growth is likely to continue in the near term; a total of 26 individuals released in 2016 and 2020 for population supplementation exerted a significant promoting effect on population recovery; flood-season hydrological fluctuations had a significant negative impact on the population, which could be effectively alleviated by targeted conservation measures; the combination of the two methods significantly improved the accuracy and reliability of parameter estimation. In summary, the Milu deer population in Dongting Lake has been successfully reconstructed under the synergistic effect of natural adaptation and targeted conservation measures. This study provides a case reference for the rewilding and recovery of endangered large ungulates in wetland ecosystems, and also serves as a typical successful example for implementing nature-based solutions to protect endangered species and biodiversity.

## Simple Summary

Milu deer (*Elaphurus davidianus*) is an endemic species and a Class I protected animal in China. According to the IUCN Red List of Threatened Species, this species is listed as Extinct in the Wild (EW). Since its natural dispersal to Dongting Lake in 1998, the species has formed the world’s only naturally dispersed and naturally rewilded population. Based on 20 years of synchronous survey data from 2006 to 2025, this study systematically analyzed the dynamic characteristics of this population using population growth models and parameter estimation methods such as Bayesian inference and nonlinear least squares (NLS). We clarified its growth patterns, the effects of population supplementation, the impacts of hydrological factors, and the effectiveness of the applied methods, providing a typical case and scientific reference for the rewilding and recovery of endangered large ungulates in wetland ecosystems.

## 1. Introduction

The conservation of endangered species is a global concern, with intensive efforts devoted to protecting and restoring their populations [1,2]. Reintroduction of species is one of the most important approaches for endangered species conservation and the restoration of their ecosystems [3]. Reintroduction refers to the intentional movement and release of an organism into its indigenous range from which it has disappeared, aiming to re-establish a viable population of the focal species within its indigenous range [3]. Species reintroductions play a crucial role in rewilding by restoring focal species’ wild populations, ecosystem processes, and restoring degraded ecological functions [4]. The success of wild population re-establishment is closely tied to species reintroduction and rewilding. Species reintroduction is a key active intervention that directly replenishes populations of endangered or locally extinct species in their native habitats. Rewilding focuses on restoring species’ independent survival abilities, such as foraging, predation, reproduction, and predator avoidance, while re-establishing natural ecological processes. These measures create a sustainable habitat for reintroduced species, facilitating their smooth integration into ecosystems and the full exertion of core ecological functions such as trophic cascades [4,5,6].

Since the initiation of species reintroduction programs in the 19th century, over 350 species worldwide have undergone reintroduction efforts [3], encompassing taxonomic groups including amphibians, reptiles, birds, and mammals. Notably, numerous iconic successful cases have been recorded, such as the Columbia Spotted Frog (*Rana luteiventris*), American alligator (*Alligator mississippiensis*), Northern Bald Ibis (*Geronticus eremita*), and Przewalski’s Horse (*Equus ferus przewalskii*) [7,8,9,10], which have successfully reestablished self-sustaining wild populations and provided valuable insights for the conservation of endangered species [11]. Since the initiation of the Milu deer (*Elaphurus davidianus*) reintroduction program in 1985 [12], China has successfully implemented reintroduction and rewilding initiatives for several endangered species, including Przewalski’s horse (*Equus ferus przewalskii*), giant panda (*Ailuropoda melanoleuca*), crested ibis (*Nipponia nippon*), and Chinese alligator (*Alligator sinensis*). These efforts have led to the successful restoration of wild populations and the rehabilitation of degraded habitats, establishing exemplary models for global endangered species conservation [13,14,15,16].

The Milu deer is a species endemic to China and a Class I National Key Protected Wild Animal. It has been listed as Extinct in the Wild (EW) on the IUCN Red List of Threatened Species. Since the implementation of the reintroduction program in 1985, a total population of approximately 9000 individuals (including both captive and wild populations) has been established in China by 2020. Among these, the wild population accounts for about 2765 individuals, which are mainly distributed in Shishou of Hubei Province, Dongting Lake of Hunan Province, and Dafeng and Yancheng of Jiangsu Province [17,18]. The Dongting Lake Milu deer population originated in 1998, when a catastrophic flood hit the Yangtze River Basin and damaged the fencing of the Shishou Milu deer National Nature Reserve in Hubei Province (SMN). Twenty-three Milu deer individuals swam across the Yangtze River, and some of them reached the East Dongting Lake National Nature Reserve (EDL) on the southern bank of the river [18]; this group successfully naturalized and developed into a wild population of 113 individuals by 2015 [19]. By 2025, the population size of the Dongting Lake Milu deer has reached 400 individuals, which are primarily distributed in the East Dongting Lake and have expanded westward to the West Dongting Lake and southward to the South Dongting Lake.

Located on the southern bank of the Yangtze River, Dongting Lake is China’s second-largest freshwater lake and an internationally important wetland. It once served as a primary distribution area for the Milu deer. However, due to large-scale historical reclamation, frequent floods, and overhunting, the distribution range and population of Milu deer gradually contracted, leading to the extinction of many large mammals (e.g., Milu deer) centuries ago [20]. Although no active Milu deer reintroduction initiatives were implemented in the Dongting Lake region prior to 2016, the catastrophic flood that ravaged the Yangtze River Basin in 1998 led to the escape of a subset of Milu deer from the adjacent SMN into EDL, where they underwent natural rewilding and established a local wild Milu deer population. Additionally, two batches of Milu deer, totaling 26 individuals, were released directly into the wild of EDL in 2016 and 2020, respectively. With no extensive human intervention—including captive breeding and adaptive rewilding training—being applied during the release process, these individuals were introduced straight into the wild, thereby forming a representative case of a fully self-sustaining wild population developed via unassisted natural rewilding in China [21]. In this study, the term “natural rewilding” refers to the spontaneous establishment of a self-sustaining wild population through natural dispersal and subsequent in situ reproduction, without active reintroduction, captive breeding, or pre-release adaptive training; the two direct-to-wild releases in 2016 and 2020 are therefore regarded as targeted conservation supplementation rather than reintroduction proper.

Population dynamics characterizes the temporal variations in population abundance and spatial distribution of wildlife [22,23]. Clarifying such ecological mechanisms helps to reveal how biological traits and environmental factors jointly drive long-term demographic changes and range shifts [24,25]. Quantitative analysis of population dynamics enables the estimation of intrinsic growth rates, the determination of habitat carrying capacity, and the identification of density-dependent regulatory processes, thereby providing fundamental evidence for scientific conservation management [26]. For reintroduced and naturally rewilded endangered species such as Milu deer, exploring both numerical and spatial dynamic patterns is essential to evaluate wild adaptation effects, interpret natural colonization processes, and optimize targeted population restoration strategies [21,27].

Existing studies on Milu deer reintroduction predominantly focus on survival performance, behavioral adaptation, and habitat suitability [12,18,28,29,30]. In contrast, quantitative research on long-term population dynamics, including the intrinsic growth rate, environmental carrying capacity, and density-dependent regulatory mechanisms, remains insufficient [17]. This knowledge gap is particularly prominent for naturally established wild populations without captive breeding and anthropogenic intervention. The Dongting Lake Milu deer population differs distinctly from conventional artificially reintroduced populations; it originated from natural colonization triggered by flood events, supplemented by additional wild releases without pre-release adaptive training, thereby forming a unique naturally established wild population. To date, its long-term growth trajectories, density-dependent demographic patterns, and ecological responses to wetland hydrological fluctuations have not been systematically quantified via modeling, which restricts the development of targeted, dynamic-based conservation and restoration strategies.

While several studies have been conducted on the Milu deer population in Dongting Lake, these investigations have primarily focused on reintroduction feasibility, short-term population surveys, and behavioral ecology [18,29,30,31]. Although some studies have documented that the population growth curve is sigmoidal in shape [19,21], they did not specify the particular growth model employed (e.g., Logistic, Gompertz, or Richards model) or estimate its parameters. This further exacerbates the aforementioned knowledge gap, particularly for a fully naturally rewilded wild Milu deer population in Dongting Lake. Drawing on synchronized survey data of the wild Milu deer population in Dongting Lake spanning the period 2006–2025, this study develops a population growth model to comprehensively characterize and analyze population dynamics. Specifically, this research addresses three core scientific questions: (1) What are the key population characteristics and temporal dynamics of the Dongting Lake Milu deer population? (2) To what extent do hydrological conditions and population supplementation influence the population’s dynamics? (3) What targeted, science-based strategies can best support the active restoration of this wild Milu deer population? Accordingly, this study tested three hypotheses: (i) the population growth follows a logistic trajectory approaching a finite carrying capacity; (ii) population supplementation is positively associated with the population growth rate; and (iii) flood-season water extent is negatively associated with population size.

The findings provide a scientific basis for the restoration and conservation of the wild Milu deer population in Dongting Lake and offer valuable insights for the reintroduction of other endangered species.

## 2. Materials and Methods

### 2.1. Study Area

**Figure 1:**
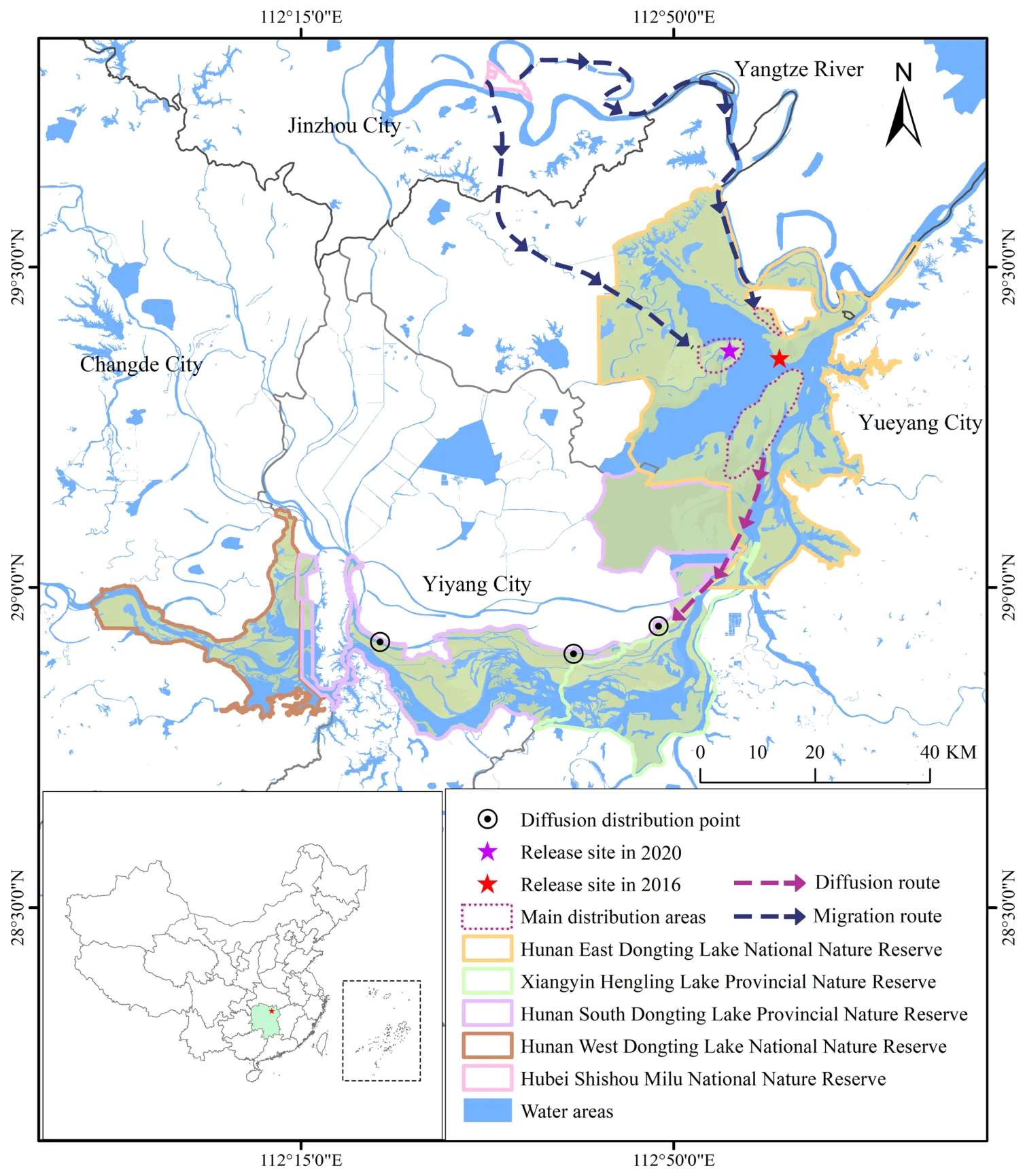
Study area of Dongting Lake.

Dongting Lake, China’s second-largest freshwater lake, is situated in northeastern Hunan Province along the southern bank of the middle Yangtze River; the lake extends between latitudes 28°44′–29°35′ N and longitudes 111°53′–113°05′ E, with a soil surface elevation ranging from 28 m to 35 m above sea level [32]. As a typical freshwater lake wetland ecosystem, it features distinct seasonal hydrological dynamics: the wet period lasts from May to October, accounting for approximately 70% of the annual mean rainfall (1429 mm), with an average water surface area of 2025 km^2^ [33]; the dry period spans November to April of the following year, with an average water surface area of 1002.7 km^2^ over the same period [34]. The wetland encompasses six key habitat types, including open water, mudflats, Carex marshes, reed marshes, forests, and other areas, forming a continuous hydrological gradient from terrestrial shrublands and meadows to shallow littoral zones and open lake waters [35]. Notably, the region is a critical habitat for overwintering migratory water birds and serves as an ideal living environment for Milu deer due to its abundant food resources, sufficient water supply, and suitable shelter conditions [36]. Currently, four natural protected areas have been established, including the EDL and West Dongting Lake National Nature Reserves (WDL), as well as the South Dongting Lake (SDL) and Hengling Lake Provincial Nature Reserves (HLN). To protect the Dongting Lake wetlands, Milu deer and their habitats, a series of initiatives including the integrated ecological restoration of mountains, rivers, forests, farmlands, lakes, grasslands and deserts, as well as habitat rehabilitation projects, have been successively implemented (e.g., restoring lake floodplains above flood level as flood refuges for Milu deer) [37] (Figure 1).

### 2.2. Animals and Data Collection

The subjects of this study were naturally rewilded wild Milu deer populations in Dongting Lake, including a wild Milu deer population that naturally rewilded after dispersing from SMN to EDL in 1998, as well as a wild population of 26 introduced Milu deer that were translocated and naturally rewilded in the wild. Among them, 16 individuals (5♂, 11♀) were translocated from Dafeng Milu Deer National Nature Reserve (Jiangsu Province) in 2016, and 10 individuals (6♂, 4♀) were translocated from Beijing Nanhaizi Milu Deer Park in 2020.

From November to December each year during 2006–2019, field surveys were mainly carried out in the EDL. Survey personnel were from the EDL and Hunan Academy of Forestry. Each year, 10–15 survey personnel (min–max) were divided into five teams (2–3 persons per team). The direct count method was used to record Milu deer abundance, and individuals were classified by age (adult/juvenile) and sex whenever possible. No remote technologies were adopted in this phase. In the winter of 2019–2020, the water level of Dongting Lake was lower than average, causing partial Milu deer dispersal from EDL toward the southwestern Dongting Lake. Consequently, annual Milu deer population surveys in November–December from 2020 to 2025 were expanded to include the Henglinghu Provincial Nature Reserve, SDL, and WDL. The annual survey team increased to 20–25 personnel (min–max), split into eight groups (2–3 persons per group): five groups for EDL and one group for each of the other three reserves. Technical staff were recruited from the four nature reserves and Hunan Academy of Forestry. Surveys were primarily conducted along lake embankments. Survey equipment included monoculars and binoculars. Starting in 2020, unmanned aerial vehicles (UAVs, drones) were applied as remote technologies for preliminary surveys to identify key Milu deer distribution areas, and additional transects were established within these key zones. Meanwhile, supplementary population information was collected through interviews with local villagers and telephone reports from residents when Milu deer entered farmlands. The reported Milu deer population values represent the maximum number of individuals directly counted in each annual winter survey; no average values were calculated from repeated surveys within a single year.

Previous studies have indicated that, influenced by the unique hydrological characteristics of Dongting Lake, habitat reduction caused by flooding and drowning deaths of Milu deer are the primary driving factors constraining population dynamics in this region [19,21]. This study selected the maximum water surface area during the flood season (July) as the core hydrological indicator and conducted correlation analysis with the Milu deer population size. Data were obtained from the scientific literature (2006–2020) and the China Hydrological Yearbook (2021–2025) [38,39,40,41,42,43,44]. China Hydrological Yearbook 2021 to 2025 (published 2022–2026), covering hydrological records during 2021–2025.

### 2.3. Data Statistical Analysis

All statistical analyses were performed using SPSS 26 (IBM Corp., Armonk, NY, USA) and Microsoft Office Excel 2010 (Microsoft Corporation, Redmond, WA, USA). Curvilinear regression analysis was used to fit population dynamics curves, with eight candidate models evaluated (Linear, Quadratic, Cubic, Power, Exponential, Growth, S, and Logistic). The optimal model was selected based on the highest coefficient of determination (R^2^), ANOVA F-test value, statistical significance (*p* < 0.0001), and visual inspection of scatter plots. According to the regression results, the exponential and logistic models were chosen for further modeling.

Using R software (version 4.2.1) [45], we applied Bayesian inference to estimate parameters of the logistic growth model, including carrying capacity (*K*), intrinsic growth rate (*r*), and intercept (*a*) [46,47]. The Metropolis–Hastings MCMC algorithm was employed with uniform priors (*K*: 500–1000; *r*: 0.05–0.5; *a*: 1–8) and heteroscedastic errors. Posterior means, medians, and 95% credible intervals were reported after 100,000 iterations (20,000 burn-in).

Meanwhile, the Nonlinear Least Squares (NLS) method was used to calculate the same parameters (*K*, *r*, *a*) of the logistic model and conduct a comparative analysis with the estimates obtained from Bayesian inference [48,49,50,51]. Subsequently, exponential and logistic growth models were constructed to simulate population dynamics under scenarios with and without population supplementation.

To assess population supplementation and no-supplementation scenarios, the formula used to calculate population size under no supplementation is: N_ns_ = N_obs_ − A − (A_f_ × R_b_ × S_f_), where N_ns_ is the population size under no supplementation, N_obs_ is the observed value, A is the number of supplemented individuals, A_f_ is the number of supplemented adult females, R_b_ is the breeding participation rate, and S_f_ is the survival rate of newborn fawns.

To evaluate how annual reproductive output (*N*) responds to variations in the female reproductive participation rate (*p*) and the fawn survival rate (*s*) under the supplemented and non-supplemented scenarios, we performed a one-at-a-time (OAT) sensitivity analysis, in which only one parameter was perturbed at a time while the others were held constant. The sensitivity index (*S*) was calculated as *S* = (Δ*N*/*N*_0_)/(Δ*x*/*x*_0_) [52], where Δ*x*/*x*_0_ is the relative change in the target parameter from its baseline value *x*_0_ and Δ*N*/*N*_0_ is the corresponding relative change in annual reproductive output from its baseline value *N*_0_.

To examine the relationship between population and maximum surface water area, while ensuring statistical power and reliability, we used the Pearson correlation analysis method and applied the Shapiro–Wilk test to verify the normality of the population size and maximum surface water area data required for Pearson correlation analysis [53]; the test results showed that the population size and the maximum water area met the normality assumption for Pearson correlation analysis. Therefore, Pearson correlation analysis was used to examine the linear relationship between population size and maximum surface water area, with a significance level set at *p* < 0.05 [54,55].

Furthermore, due to the unique natural and hydrological characteristics of Dongting Lake, it is difficult to count births and deaths in the wild. Therefore, the annual population growth rate was calculated using the following formula: Annual population growth rate = N_t_ − N_t−1_ − R_t_/N_t−1_ (N_t_ is the population size at the end of year t, N_t−1_ is the population size at the end of year t − 1, R_t_ is the number of reintroduction individuals in year t).

### 2.4. Population Growth Models

According to the regression results of the population growth curve, in this study, the exponential and logistic growth models were adopted, represented by the following differential equations [56,57,58,59]:

(1) $$\frac{\mathrm{dN}}{\mathrm{dt}}=\mathrm{rN}$$

(2) $$\frac{\mathrm{dN}}{\mathrm{dt}}=\mathrm{rN}(1-\frac{N}{K})\;$$

The integral forms of these differential equations are as follows:

*N_t_* = *N*_0_*e^rt^* (3)

(4) $$N_{t}=\frac{K}{\left[1+e^{a-\mathrm{rt}}\right]}$$

where $\frac{\mathrm{dN}}{\mathrm{dt}}$ is the rate of change in population size, *N* is the population size, *e* is the base of the natural logarithm, *r* is the intrinsic growth rate, *t* is time, *N_t_* is the population size at time *t*, *N*_0_ is the initial population size, *K* is the carrying capacity, and *a* is the intercept, representing the relative position of the curve from the origin.

### 2.5. Estimation Parameters of Logistic Model

The parameters (*K*, *r*, *a*) of the logistic model were estimated using Bayesian inference and the nonlinear least squares method.

#### 2.5.1. Bayesian Inference Method

The posterior distributions of parameters were derived based on Bayes’ theorem:

*P*(*K*,*r*,*a*∣ *N_t_*)∝*P*(*N_t_*∣ *K*,*r*,*a*) ⋅ *P*(*K*,*r*,*a*) (5)

where *P*(*K*, *r*, *a*∣*N_t_*) is the posterior distribution of parameters, *P*(*N_t_*∣*K*, *r*, *a*) is the likelihood function (assuming a normal distribution for observation errors), and *P*(*K*, *r*, *a*) is the prior distribution of parameters (Uniform for (*K*), Normal for (*r*), and LogNormal for (*a*)).

#### 2.5.2. Nonlinear Least Squares Method

The NLS method estimates parameters by minimizing the sum of squared residuals between observed and predicted population sizes, and its core formula is as follows:

(6) $$\hat{K},\hat{r},\hat{a}=\arg\underset{\left\{K,\;r,\;a\right\}}{\min}\sum_{t=1}^{T}{\left(N_{\mathrm{obs}}-\frac{K}{1+e^{a-\mathrm{rt}}}\right)}^{2}$$

where *T* is the total number of observations, *K* (carrying capacity), *r* (intrinsic growth rate), and *a* (growth constant) are the parameters to be estimated, and the sum term represents the sum of squared residuals between observed values (*N_obs_*) and logistic model predicted values (*N_t_*).

### 2.6. Population Growth Models Construction

Based on the logistic model parameters (*K*, *r*, *a*) estimated by Bayesian inference and nonlinear least squares (NLS), the parameter values were substituted into Equation (4) to establish logistic growth models for the Milu deer population in Dongting Lake. The models were used to analyze population characteristics and dynamics, and to simulate population trends under scenarios with and without population supplementation. The exponential population growth model and corresponding parameters were derived via exponential regression analysis of population abundance curves generated from synchronous population surveys.

## 3. Results

### 3.1. Population Dynamics

**Figure 2:**
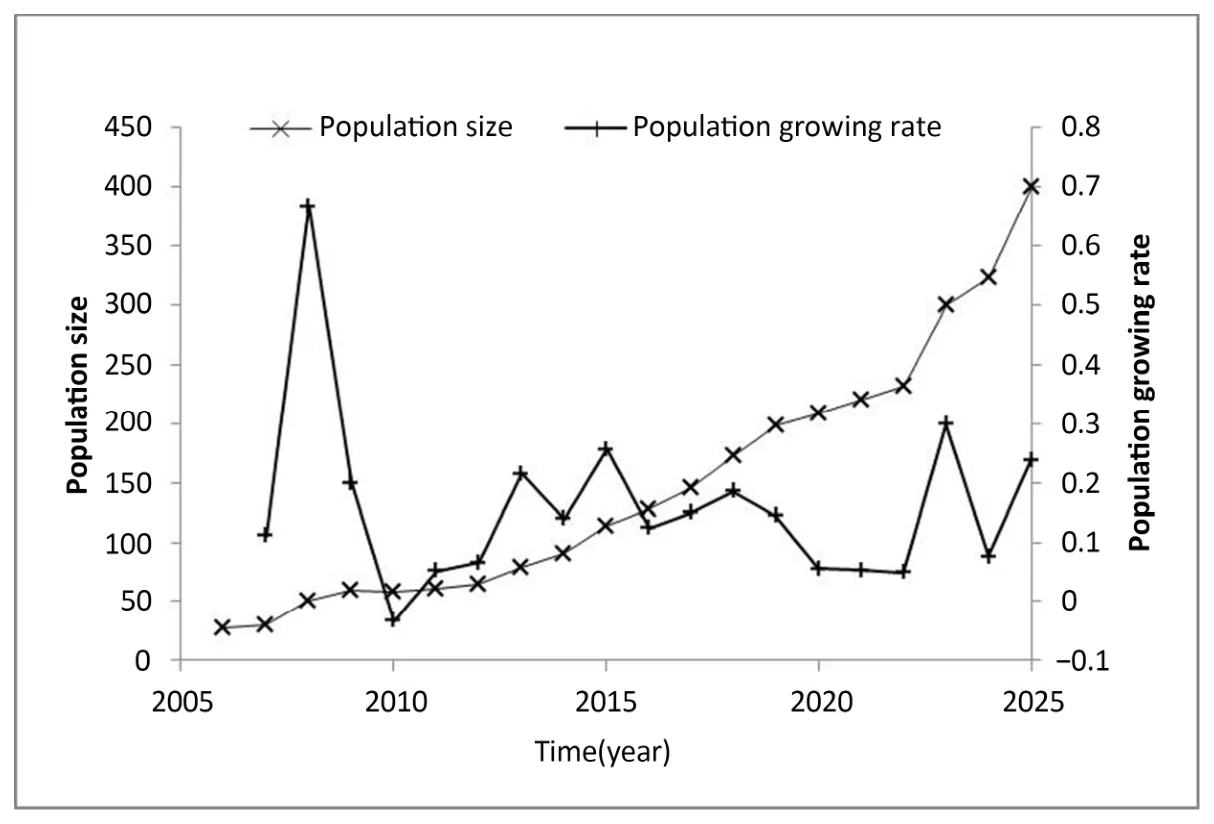
Population size and annual growth rate of the naturally rewilded Milu deer in Dongting Lake, 2006–2025. Population size is indicated by ×(left *y*-axis), and annual population growth rate by +(right *y*-axis).

The population of Milu deer in Dongting Lake increased from 27 individuals in 2006 to 400 individuals in 2025, with an average annual increase of 19.63 ± 4.58 individuals and an average annual growth rate of 0.16 ± 0.03 across the whole study period. During 2006–2015, the average annual population increase was 9.55 ± 2.65 individuals, with an average annual growth rate of 0.19 ± 0.06. During 2016–2025, the average annual population increase rose to 28.70 ± 7.64 individuals, while the average annual growth rate was 0.14 ± 0.03 (Figure 2). Overall, the Milu deer population showed a continuous increasing trend, with a higher absolute annual increment but a slightly lower relative growth rate in the second half of the monitoring period.

At present, the population of Milu deer is predominantly concentrated in the western and central parts of the EDL. An expansion trend has been observed, with the population gradually diffusing towards the HLN, SDL, and WDL. Due to the isolation of Dongting Lake by the Yangtze River, surrounding embankments, highways, and human activity areas, it functions as a relatively independent habitat. The migration and diffusion of the Milu deer population are restricted to short-distance horizontal movements within the Dongting Lake wetland area, occurring primarily during the winter low-water season.

### 3.2. Population Growth Model

**Figure 3:**
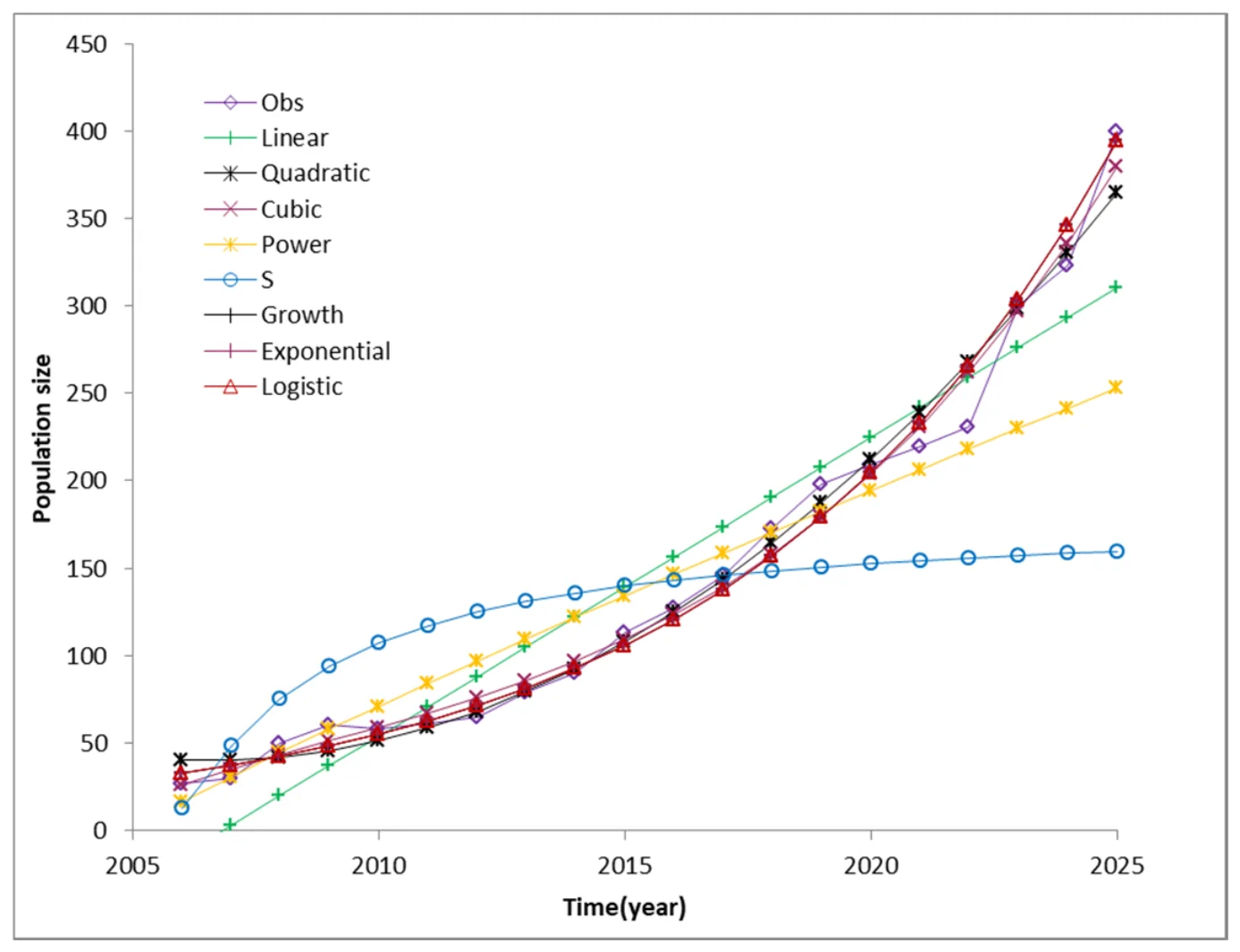
Fitted curves of eight growth models (Linear, Quadratic, Cubic, Power, Exponential, Growth, S-curve, Logistic) and observed population size of Milu deer in Dongting Lake over a 20-year monitoring period. Purple diamonds represent actual population survey data (Obs), and colored lines represent predicted values from each model. Among them, the Growth, Exponential, and Logistic models show the highest goodness of fit to the observed population growth trajectory.

Curvilinear regression analysis based on the Milu deer population size from 2006 to 2025 showed that all fitted models were highly significant (*p* < 0.01). The Growth, Exponential, and Logistic models had the highest coefficient of determination (R^2^ = 0.98) and ANOVA F value (F = 912.18) (Table 1, Figure 3). Since the Growth model and Exponential model are mathematically equivalent, and the regression curves of the exponential and logistic models are closer to the curve of observed values, the Exponential and Logistic growth models were finally used in this study.

### 3.3. Parameter Estimation

**Figure 4:**
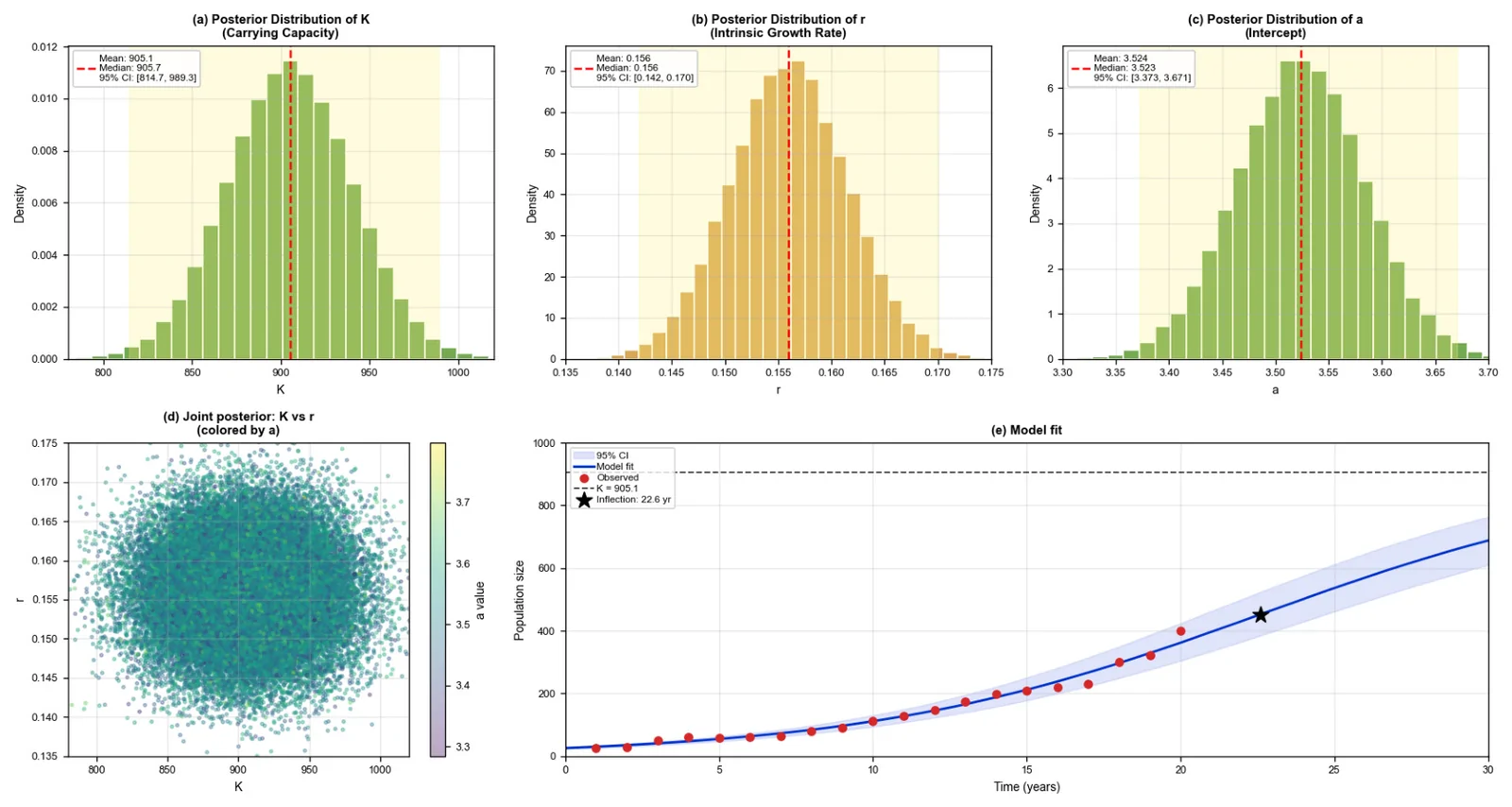
Bayesian inference for parameter estimation and fitting of the logistic growth model. (**a**) posterior distribution of carrying capacity *K*; (**b**) posterior distribution of intrinsic growth rate *r*; (**c**) posterior distribution of intercept *a*; (**d**) joint posterior distribution of *K* and *r*, colored by *a*; (**e**) logistic model fit to observed data, with uncertainty bands and inflection point.

Based on Bayesian inference, the logistic growth model provided reliable estimates of population parameters. The posterior mean carrying capacity *K* was 905.10 (median = 921.40, 95% CI = 730–997), and the posterior mean intrinsic growth rate *r* was 0.156 (median = 0.156, 95% CI = 0.147–0.168). The intercept *a* had a posterior mean of 3.524 (median = 3.534, 95% CI = 3.33–3.68). Strong correlations were observed among *K*, *r*, and *a* in the joint posterior distribution. The maximum a posteriori logistic growth curve fitted the observed population data well, with the 95% credible interval indicating prediction uncertainty. The Bayesian model showed excellent goodness-of-fit, with R^2^ = 0.980 and RMSE = 13.59. The inflection point of the population growth trajectory was estimated at 22.6 years, corresponding to a population size of 452.6 individuals (Figure 4).

Nonlinear least squares (NLS) fitting was performed using SPSS software, with population size as the dependent variable and time as the independent variable, to fit the logistic growth model. Based on the published intrinsic growth rate *r* (0.15–0.19) of Milu deer from the logistic population model [60], parameter constraints were defined within this interval. Combined with the intercept parameter (*a*) obtained from Bayesian inference, we imposed the following bounds during model fitting: 0.1 < *r* < 0.17 and 1.0 < *a* < 3.6. The initial values were set as *K* = 600.00, *a* = 1.00, and *r* = 0.10. Sequential Quadratic Programming was adopted, and convergence was achieved after 15 iterations, with a residual sum of squares (RSS) of 3520.15 (df = 17), a root mean square error (RMSE) of 14.39, and a coefficient of determination (R^2^ = 0.984). The final convergent parameters were *K* = 984, *a* = 3.60, and *r* = 0.154.

Compared with the Bayesian inference, parameter estimates from NLS showed slight differences. The intrinsic growth rate *r* decreased from 0.156 to 0.154, with a relative difference of 1.28%, indicating near equivalence. The carrying capacity *K* increased from 905.1 to 984, an 8.72% rise, and this NLS estimate lies within the credible interval of the Bayesian *K*. Parameter *a* increased from 3.524 to 3.60, with a relative increase of 2.16%, showing good agreement.

The two methods exhibited good consistency in estimates of r and a, and NLS results serve as a helpful auxiliary reference for Bayesian inference. The NLS (*K* = 984) lies within the Bayesian credible interval for *K*, lending support to this carrying capacity estimate. However, we acknowledge inherent limitations in the *K* estimate: the population time series has not reached the saturation plateau of the logistic curve, and exponential and logistic models produce nearly identical fits to our observed data, which introduces uncertainty into *K* estimation. Nevertheless, the upper bound for *a* was predefined before fitting, whereas *K* was estimated freely by the model, and the fitted *a* reached this preset boundary. The Bayesian model had high predictive performance (R^2^ = 0.980, RMSE = 13.59), comparable to NLS (R^2^ = 0.984, RMSE = 14.39), with a slightly smaller RMSE.

### 3.4. Population Growth Models Construction

Parameter estimation results of the logistic population growth model based on Bayesian inference were obtained as *K* = 905.1, *a* = 3.52, and *r* = 0.156. These parameters were substituted into Equation (4) to establish the logistic growth model for Milu deer: *N_t_* = 905.1/1 + exp(3.52 − 0.156*t*).

**Figure 5:**
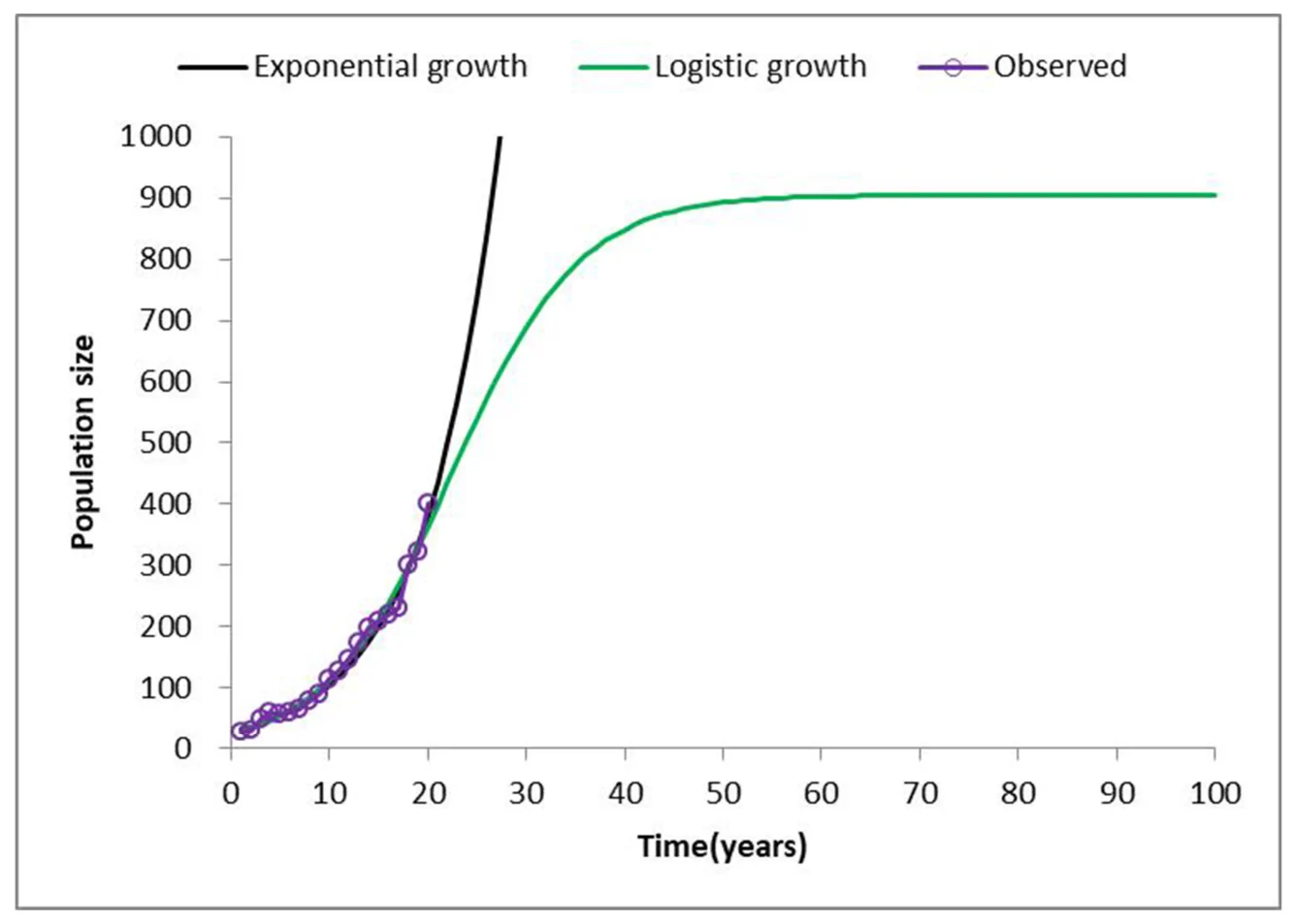
Comparison of observed Milu deer population dynamics with exponential and logistic growth model predictions over a 100-year time horizon. Three population trajectories are presented: (1) the exponential growth model (black solid line), representing density-independent, unrestricted growth under ideal conditions, which shows continuous, accelerating increase in population size; (2) the logistic growth model (green solid line), a density-dependent, sigmoidal (S-shaped) curve that exhibits rapid initial growth, followed by a gradual deceleration and stabilization at the estimated carrying capacity (*K* ≈ 900 individuals) after approximately 50 years; and (3) observed population data (purple open circles), which closely follows the logistic growth pattern during the early growth phase (0–20 years), reflecting natural population dynamics constrained by environmental resources. This comparison illustrates the divergence between theoretical exponential growth and biologically realistic growth regulated by carrying capacity.

The observed population size data were fitted to the exponential growth curve to obtain their exponential growth models. The formula of the exponential growth model for the Milu deer is *N_t_* = 28.39exp(0.13*t*) (Figure 5).

Model simulation results indicated that the population of Milu deer in Dongting Lake exhibited exponential growth in the first 22.6 years (*a*/*r*), followed by a gradual slowdown in population growth, reaching the peak of the carrying capacity around year 50 (Figure 5). Results from fitting 20 years of observed data showed that the exponential growth model yielded a coefficient of determination R^2^ = 0.99, root mean square error RMSE = 30.27, mean absolute error MAE = 22.65, and mean residual (systematic bias) = 2.82. Paired *t*-tests demonstrated no significant difference between exponential predictions and observations (*p* = 0.31 > 0.05). The logistic growth model achieved R^2^ = 0.98, RMSE = 18.30, MAE = 13.80, and mean residual (systematic bias) = −0.90, with no significant difference from observed values (*p* = 0.77 > 0.05). Both models showed good agreement with the observed population dynamics, while the logistic model exhibited smaller errors and no obvious systematic bias, indicating better overall performance.

### 3.5. Simulation Analysis of Population Supplementation

To examine the effect of population supplementation, we assumed all released individuals survived until 2025, with an average lifespan of 13–15 years; the breeding participation rate of adult females was 0.67, and the survival rate of newborn fawns was 0.78 [19]. Based on the formula N_ns_ = N_obs_ − A − (A_f_ × R_b_ × S_f_), we calculated the population size under the no-supplementation scenario, the population size was reduced by 22 individuals annually (e.g., N_obs_ = 127, A = 16, A_f_ = 11, R_b_ = 0.67, S_f_ = 0.78, N_ns_ = 22) during 2016–2019 and by 34 individuals annually (e.g., N_obs_ = 209, A = 11, A_f_ = 4, R_b_ = 0.67, S_f_ = 0.78, cumulative reduction, N_ns_ = 34) during 2020–2025. Simulation analyses showed that under the no-supplementation scenario, the average annual increase in the number of individuals and the average annual growth rate were 25 ± 8.7 individuals and 0.13 ± 0.04 from 2016 to 2025, respectively; the average annual increase in the number of individuals and annual growth rate were 18 ± 4.87 and 0.16 ± 0.04 from 2006 to 2025, respectively.

A one-at-a-time sensitivity analysis was performed to evaluate how annual reproductive output *N* responds to variations in female reproductive participation rate *p* and fawn survival rate *s*. The results are presented in Table 2. The sensitivity index *S* was 1.00 for all non-baseline scenarios. This means that the relative change in annual reproductive output is proportional to the relative change in *p* or *s*.

**Figure 6:**
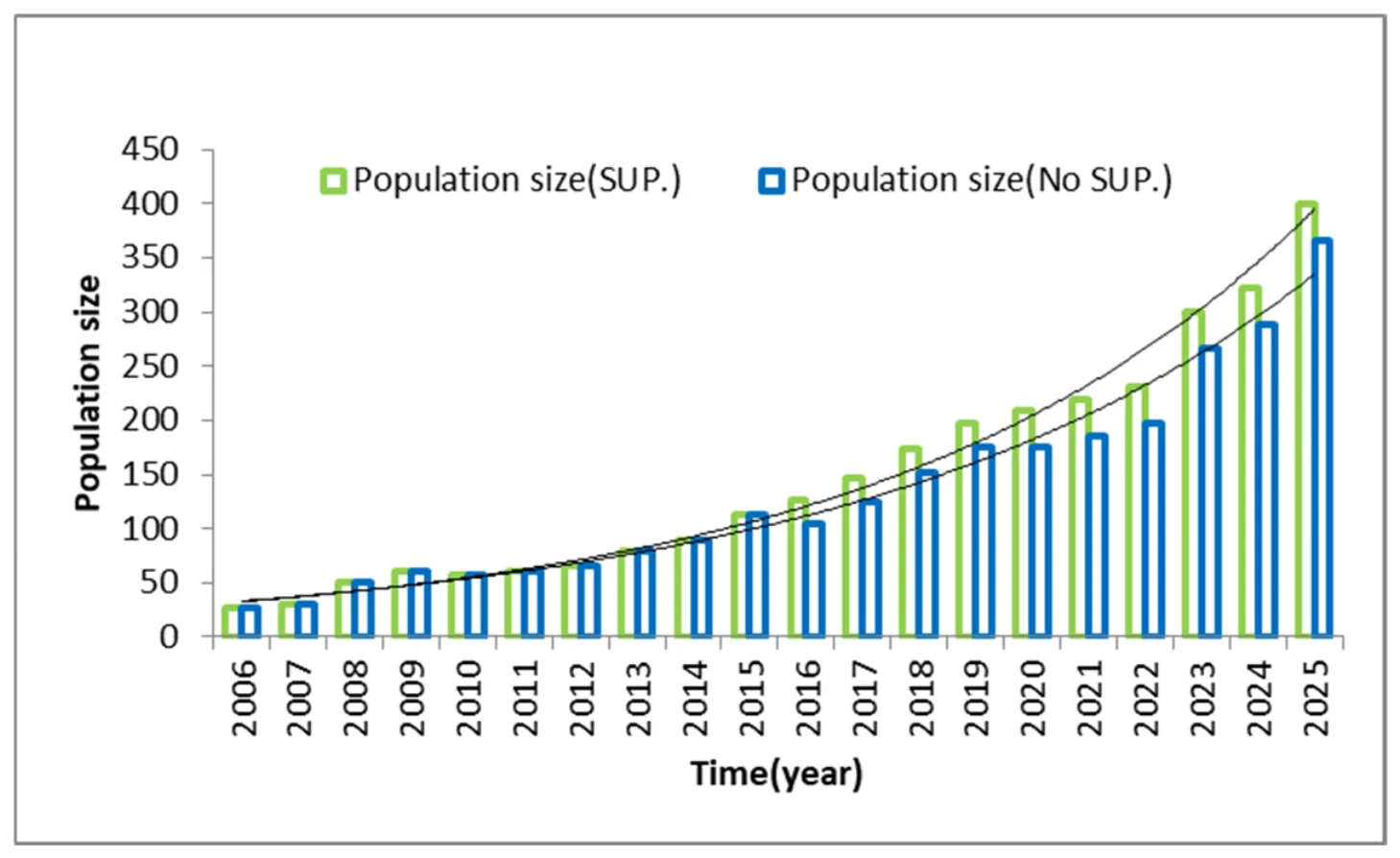
Population growth dynamics of Milu deer under supplemented and non-supplemented scenarios, 2006–2025. The supplemented population (green bars, with trend line) showed accelerated growth, reaching approximately 400 individuals by 2025. The non-supplemented population (blue bars, with trend line) exhibited a slower growth trajectory, totaling approximately 370 individuals in the same year. The widening gap between the two curves after 2015 highlights the critical role of supplementation in accelerating Milu deer population recovery. Abbreviations: SUP, supplementation scenario; No SUP, non-supplementation scenario.

Compared with the no-supplementation scenario, population supplementation significantly improved the average annual increment in population size and the average annual growth rate. A paired-samples *t*-test showed that annual population size was significantly higher under the supplementation scenario (*t* = 4.18, *df* = 19, *p* < 0.01). From 2016 to 2025, population supplementation resulted in a 14.80% relative increase in annual population increment and a 7.69% relative increase in annual growth rate compared with the non-supplementation scenario (Table 3, Figure 6).

**Figure 7:**
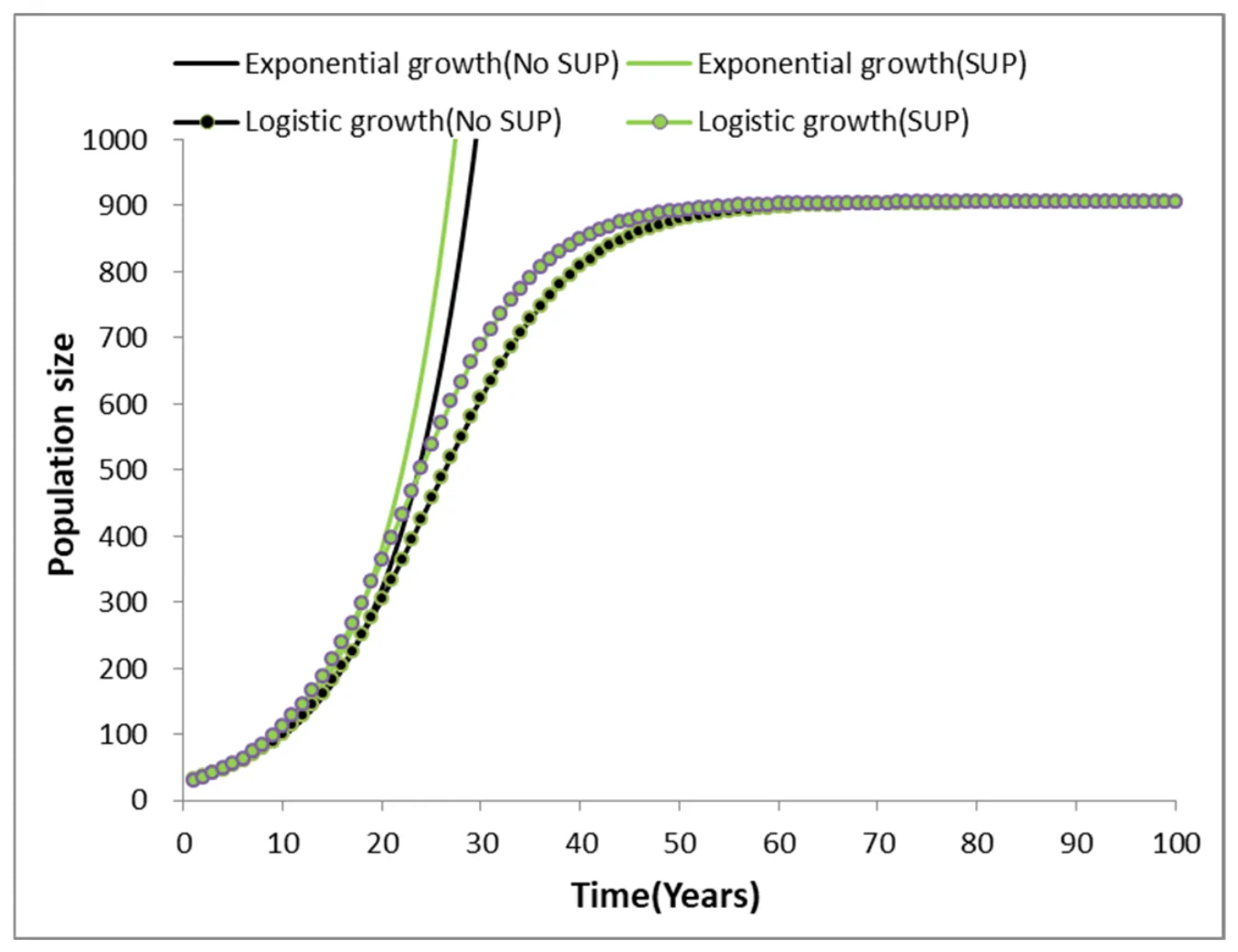
Simulated population growth of Milu deer under supplementation and non-supplementation scenarios in Dongting Lake. Population size was projected over 100 years using exponential and logistic growth models. Green lines indicate the supplementation scenario, and black lines indicate the non-supplementation scenario. Abbreviations: SUP, supplementation scenario; No SUP, non-supplementation scenario.

In the non-supplementation scenario, the exponential and logistic growth models were N_t_ = 29.1exp(0.12t) (R^2^ = 0.97) and N_t_ = 905.1/[1 + exp(3.48 − 0.14t)] (R^2^ = 0.97), respectively. Paired *t*-tests further revealed highly significant differences between predicted values with and without supplementation scenarios in both the exponential model (t = 3.90, df = 19, *p* < 0.01) and the logistic model (t = 4.24, df = 19, *p* < 0.01); the intrinsic growth rates *r* in the exponential and logistic growth models increased by 8.33% and 14.29%, respectively (Table 4, Figure 7).

The simulation analysis of population growth models shows that supplementation measures for the Milu deer population in the Dongting Lake area produced a significant increase in population growth. Under the supplementation scenario, the intrinsic growth rate *r* estimated by both the exponential growth model and the logistic growth model was higher than that under the non-supplementation scenario, by 8.33% and 14.29% (Table 4), respectively. This indicates that population supplementation elevated the population’s reproductive potential and growth momentum. It should be noted that statistically significant differences between modeled scenarios cannot independently prove that the population model is correct; such differences only represent predicted outcomes under predefined demographic assumptions.

### 3.6. Hydrological Impact on Population Dynamics

To account for the potential effects of artificial interventions, such as population supplementation and habitat improvement (e.g., restoring lake floodplains above flood level as flood refuges for Milu deer), correlation analyses were conducted across distinct time periods. Pearson correlation analysis showed that there were significant correlations between Milu deer population dynamics and the maximum water surface area, although these relationships varied considerably among different periods. The findings revealed the following: during 2006–2015 (a period without population supplementation or Milu deer refuge platforms), there was a weak to moderate negative correlation between Milu deer population dynamics and the maximum water surface area (*r* = −0.36, *n* = 10, *p* = 0.307); During 2006–2023 (a period without Milu deer refuge platforms; two population supplementation measures were implemented after 2016), a moderate negative correlation was observed between population dynamics and the annual maximum water surface area, though the relationship did not reach statistical significance (*r* = −0.39, *n* = 18, *p* = 0.110); The strongest negative correlation occurred during 2016–2023 (*r* = −0.89, *n* = 8, *p* < 0.01) and 2019–2023 (*r* = −0.91, *n* = 5, *p* = 0.032 < 0.05), even though the population was supplemented twice during this period (in 2016 and 2020); however, over the period of 2006–2025, no significant correlation was observed between population dynamics and the maximum water surface area (*r* = −0.08, *n* = 20, *p* = 0.737) (Table 5).

**Figure 8:**
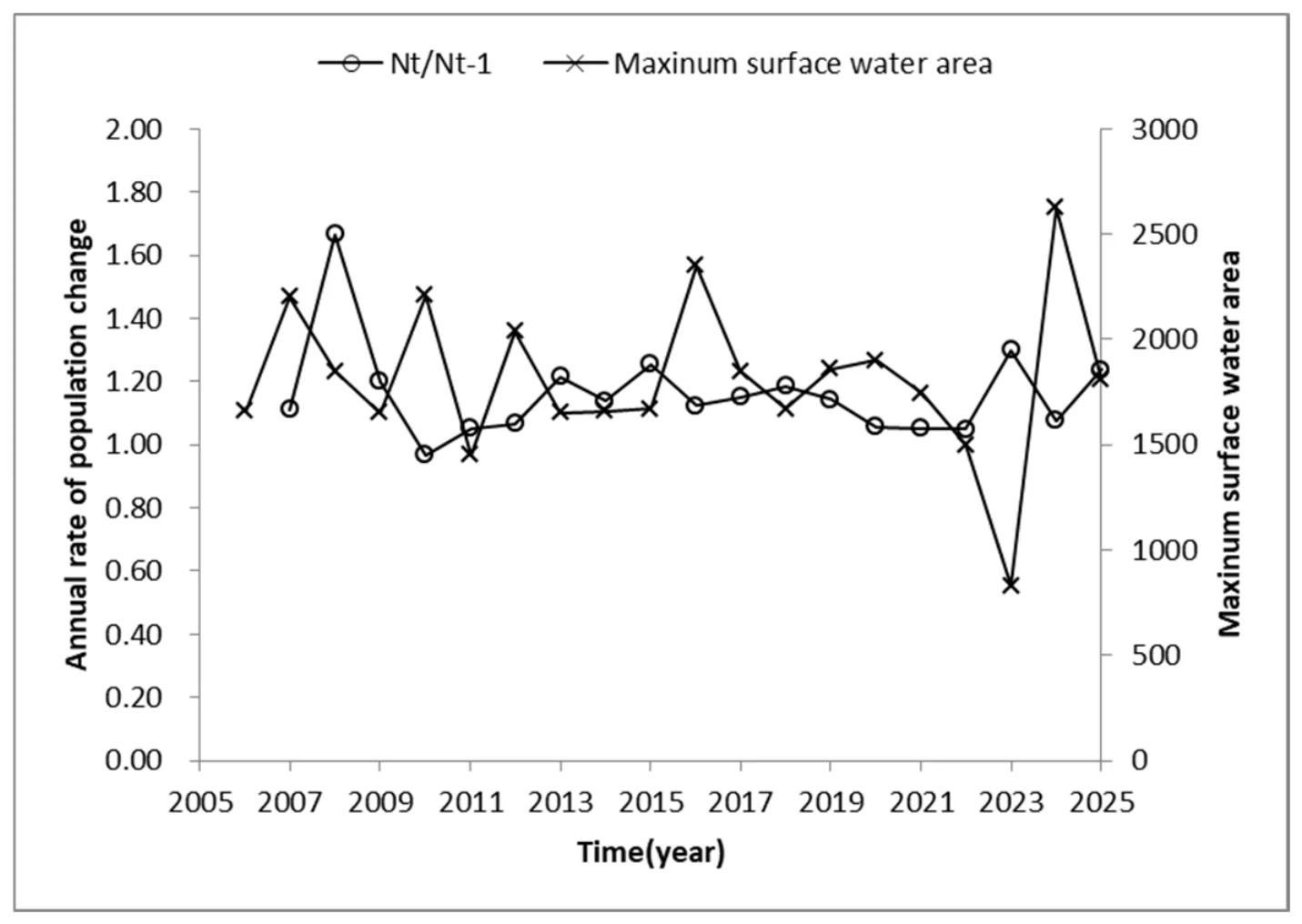
Annual rate of population change in Milu deer and maximum surface water area in Dongting Lake, 2006–2025. The annual rate of population change in Milu deer (N_t_/N_t−1_) (○, left *y*-axis) exhibited marked interannual fluctuations across the study period, where a larger maximum surface water area (×, right *y*-axis, km^2^) tended to correspond to a reduced annual rate of population change in the subsequent year, with an abrupt drop in surface water area in 2023.

**Figure 9:**
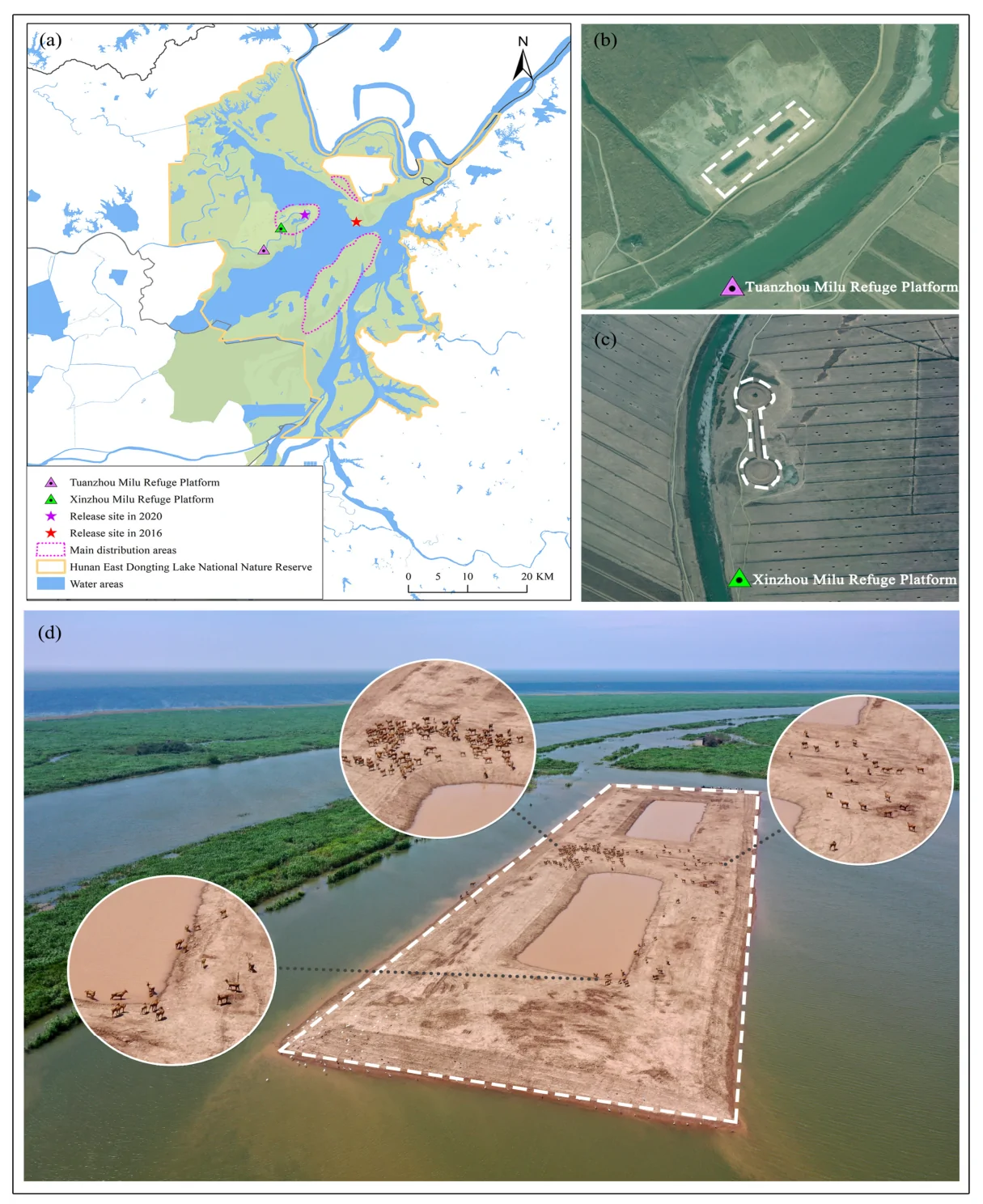
Location map of Milu deer refuge platforms in the East Dongting Lake National Nature Reserve and photographs of Milu deer taking shelter from floods on the platforms during the 2024 flood season. (**a**) Location map of Milu deer refuge platforms; (**b**) morphology of the Tuanzhou Milu deer refuge platform (12 ha) and (**c**) Xinzhou Milu deer refuge platform (2.2 ha); (**d**) Milu deer taking shelter from floods on the refuge platforms during the 2024 flood season.

The divergent relationships between population dynamics and maximum surface water area across different periods were likely strongly associated with the implementation of anthropogenic interventions during the study. From 2006 to 2015, in the absence of population supplementation, the expansion of maximum surface water area had a negative effect on population growth. From 2006 to 2023, despite the implementation of population supplementation, the lack of operational Milu deer refuge platforms meant that such interventions did not fully offset the adverse impacts of flooding. Following the operationalization of Milu deer refuge platforms in 2024, the combined effects of flood avoidance and population supplementation may have masked the negative association between the two variables. This resulted in a non-significant correlation over the entire study period from 2006 to 2025 (Table 5, Figure 8 and Figure 9).

## 4. Discussion

In this study, we constructed exponential and logistic growth models, combined Bayesian inference with nonlinear least squares (NLS) for parameter estimation, set up scenario simulations with and without population supplementation, and analyzed the correlation between hydrological factors and population size to systematically clarify the growth characteristics, core influencing factors, and actual effects of artificial protection measures for this population. Despite the numerous modifications and improvements to the logistic growth model proposed by scholars [61], both the classic model and its modified versions have certain limitations. Nevertheless, both the exponential and logistic models remain significant in population ecology and have been widely used for over a century [58,59,61]. The results of this study not only clarify the development trajectory of the Milu deer population under the background of natural rewilding, but also provide a targeted scientific basis and case reference for the reintroduction, rewilding, and recovery of endangered species, as well as the formulation of species protection strategies in wetland ecosystems, from both methodological and practical perspectives.

### 4.1. Population Growth Characteristics and Hydrological Limitations

The results of this study show that from 2006 to 2025, the Milu deer population in Dongting Lake increased from 27 to 400 individuals, with an average annual growth rate of 0.16 ± 0.03, and the fitted models indicate that rapid population growth is likely to continue in the near term, followed by a gradual slowdown as the population approaches the estimated carrying capacity. This characteristic is highly consistent with the development background of natural rewilding of this population. The intrinsic growth rates fitted by the exponential and logistic models were 0.13 and 0.16, respectively. The carrying capacity *K* estimated by Bayesian inference was 905.1 (95% CI: 730~997), and that estimated by the NLS method was 984.

The two estimates are mutually consistent; the NLS estimate falls within the Bayesian credible interval, although both should be interpreted with caution because the population has not yet reached the saturation plateau of the logistic curve and *K* remains weakly identifiable. Both suggest the carrying capacity of Dongting Lake wetland for the Milu deer population. Compared with populations under intensive human intervention in Shishou, Dafeng, and other regions, the growth rate of the Dongting Lake Milu deer population is relatively low [19], and the major limiting factor appears to be the abiotic stress caused by seasonal hydrological fluctuations in the lake area. As a typical floodplain wetland, the high water level in Dongting Lake during the flood season will directly compress the foraging and habitat of Milu deer, even leading to individual drowning, and isolate the population into small groups, reducing the mating probability and resulting in a low conception rate. From 2016 to 2023, there was a significant negative correlation between population size and the maximum water area during the flood season (*r* = −0.89, *n* = 8, *p* < 0.01), which is consistent with an inhibitory effect of hydrological factors on population growth.

In contrast, the SMN and Dafeng nature reserves are less affected by extreme hydrological disturbances and have more human interventions, resulting in significantly improved habitat stability, which has become a key condition for their high population growth rate [12,17]. This comparison reveals the core role of habitat stability in the recovery of naturally rewilded populations of endangered species, and also indicates that the growth rules of naturally rewilded populations are more likely to be restricted by the inherent characteristics of the natural environment, and their protection strategies need to be fully adapted to the natural dynamic characteristics of the habitat.

### 4.2. Effects of Population Supplementation and the Principle of Appropriateness

Population supplementation (26 individuals released in 2016 and 2020) strongly promoted Milu deer population growth in Dongting Lake. Scenario simulations revealed that population growth curves differed extremely significantly between supplementation and non-supplementation scenarios (*p* < 0.01). It should be emphasized that this comparison relies on a modeled counterfactual scenario constructed under the assumed demographic parameters rather than on a direct observation or an experimental comparison; the results therefore support the hypothesis of a positive effect of supplementation but cannot by themselves quantify its direct causal effect. Under the non-supplementation scenario, the intrinsic growth rates of exponential and logistic models decreased by 8.33% and 14.29%, respectively, relative to the supplementation scenario, and the average annual population increment was also lower.

Sensitivity analysis (Table 2) showed that annual reproductive output changed proportionally with the reproductive participation rate of supplemented females (*p*) and fawn survival rate (*s*). This proportionality arises directly from the multiplicative model (*N* = *F* × *p* × *s*), meaning equal relative changes in *p* or *s* produce identical relative changes in reproductive output within the model. Nevertheless, this finding is model-dependent and should be interpreted cautiously because real ecological systems may contain nonlinear relationships omitted from this simplified model.

The effects of population supplementation operate in two dimensions: first, by contributing to expanding the population base; second, by promoting population recovery and laying the foundation for exponential population growth, a pattern consistent with findings from reintroduction programs for endangered species such as Przewalski’s horse and Crested Ibis [13,15]. It is worth noting that the “boosting effect” of population growth after supplementation is particularly significant in the short term, but this measure is not an unlimited protection method. Its implementation must be based on the scientific assessment of the carrying capacity. Excessive supplementation is likely to cause resource competition and habitat degradation. In the future, it is necessary to combine real-time monitoring of population density and habitat quality to achieve precise and moderate supplementation measures.

### 4.3. Population Growth Model Applicability Analysis

Bayesian inference and nonlinear least squares (NLS) were used to fit the logistic population growth model for Milu deer, and the two methods yielded generally consistent parameter estimates. The intrinsic growth rate (*r*) differed by only 1.28% between approaches, and the NLS estimates of carrying capacity (*K*) and intercept constant (*a*) were close to the posterior means from Bayesian inference; notably, the NLS-derived *K* fell within the 95% credible interval of the Bayesian *K*. The high coefficients of determination (R^2^ > 0.98) and low RMSE values indicated that both models fitted the observed population time series well, which supports the reliability of the estimated demographic parameters.

Despite the good consistency of *r* and *a*, the carrying capacity *K* showed a relatively larger relative difference (8.72%) between the two fitting methods. This uncertainty mainly arises because the field population has not yet reached the saturation plateau of the logistic curve. At the current stage, exponential and logistic models provide almost identical fits to the monitoring data, which weakens the identifiability of *K*. In addition, the integration constant *a* reached its upper preset boundary in the NLS fitting, suggesting that the preset parameter constraints may affect the optimization results and should be interpreted with caution.

Overall, NLS can act as a useful supplementary reference for Bayesian parameter inference. The Bayesian logistic model exhibited slightly better predictive performance (lower RMSE), and its posterior distribution can directly quantify parameter uncertainty via credible intervals, which is an advantage over point estimates from NLS. Considering the uncertainty of *K*, the carrying capacity estimated in this study should be regarded as a preliminary reference for habitat management rather than a fixed threshold. Long-term population monitoring is still required to further constrain the logistic curve and improve the accuracy of *K* estimation for the Milu deer population.

### 4.4. Hydrological Constraints and Synergistic Effects of Targeted Conservation Measures

Synergistic deployment of targeted conservation interventions serves as the core guarantee for mitigating natural environmental limitations and sustaining steady population growth of Milu deer in Dongting Lake, which is fully supported by correlation analyses of hydrological variables at multiple temporal scales. In the absence of targeted conservation actions from 2006 to 2015, population size exhibited a weak-to-moderate negative correlation with maximum flood-season inundation area; between 2016 and 2023, when only population supplementation was conducted without complementary habitat restoration, including Milu deer flood refuge platforms, this negative association became statistically significant (*r* = −0.89, *n* = 8, *p* < 0.01).

The launch of flood refuge platforms in 2024 created a combined synergistic effect with long-term population supplementation, eliminating the significant correlation between overall Milu deer population abundance and peak flood inundation area across the full 2006–2025 dataset (*r* = −0.08, *n* = 20, *p* = 0.737 > 0.05), a pattern consistent with a combined effect of flood avoidance and long-term population supplementation. This result shows that a single population supplementation measure cannot completely offset the negative impact of natural hydrology, while targeted conservation measures for habitat improvement (such as Milu deer refuge platforms) can directly improve the living environment of species, reduce the mortality risk of extreme hydrological events, and form a “1 + 1 > 2” synergistic effect with population supplementation, effectively offsetting the constraints of the natural environment. It should also be noted that the platforms have been operational only since 2024, so the post-implementation period is short; direct observations of deer sheltering on the platforms during the 2024 flood season (Figure 9) confirm that they were used as refuges, but longer post-implementation monitoring is required to demonstrate an effect on the dynamics of the population as a whole.

This finding reveals the conservation mechanism of naturally rewilded populations of endangered species: natural recovery is the foundation, while targeted conservation measures can accelerate the recovery process, and the synergistic implementation effect of multiple targeted conservation measures is far better than that of a single measure. The successful experience of population reconstruction for Milu deer in Dongting Lake, especially the process of natural dispersal, natural reintroduction, and rewilding, is not only in line with the principles and goals of Nature-based Solutions (NbS) under the Kunming-Montreal Global Biodiversity Framework [62,63,64], but also represents a typical successful practice of nature-based solutions.

### 4.5. Limiting Factors and Conservation Implications for Dongting Lake Milu Deer

Limited dispersal and insufficient habitat connectivity restrict Milu deer population expansion at Dongting Lake, necessitating refined conservation schemes [18,19]. Sand mining has destroyed 52 km^2^ of lake beaches and islands since 1989, impairing habitat integrity [65]. Fragmentation, dikes, and roads trap most Milu deer in East Dongting Lake with little cross-lake dispersal [19,29,30], which will intensify intraspecific competition near carrying capacity and suppress long-term population growth. Logistic models forecast saturation within 50 years; the ongoing transition from exponential to logistic growth prioritizes habitat expansion and connectivity improvement [12,21].

Under stepping-stone and dispersal theories, cross-lake ecological corridors build functional Milu deer metapopulations [66,67,68], reduce fragmentation, ease human disturbance, renovate flood refuges and enlarge suitable habitats, facilitating wide-range dispersal, inter-population gene flow and higher genetic viability [66]. The natural rewilding of this Milu deer population delivers a Chinese template for global endangered species restoration with three key insights. First, Milu deer show strong wild adaptability; the self-sustaining population naturally dispersed since 1998 validates habitat-centered protection over excessive artificial management [65]. Second, conservation measures should match population growth stages [28]; supplementation and habitat restoration drive exponential growth, whereas habitat expansion and genetic-demographic optimization sustain stable logistic growth [69,70]. Third, long-term field monitoring combined with modeling predicts population dynamics to enable adaptive management, forming a transferable framework for endangered species protection [71,72]. As a wetland flagship species, Milu deer recovery modulates vegetation and nutrient cycling and elevates wetland biodiversity and ecosystem stability [21,29,30]. Future work should advance integrated wetland restoration, alleviate sand mining, reclamation, and aquaculture disturbances, and extend these strategies to sympatric *Hydropotes inermis* [73,74]. This nature-based conservation case offers practical references for worldwide wetland biodiversity protection and species rewilding.

This study has several limitations guiding future research. First, although data on adult, fawn, and sex composition of the Milu deer population were collected, the incompleteness of these data precluded analyses using age- and sex-structured models; age and sex structure of Milu deer were not examined, despite their strong influence on population vital rates [29]. Population dynamics were therefore analyzed only with the population growth model, which does not require age and sex structure, and the results should be interpreted with certain limitations. Future studies can further collect age and sex data and use age- and sex-ratio models to interpret population structure and dynamics. Second, only hydrological factors and population supplementation were considered, while food, interspecific competition, and disease were not quantified. Subsequent studies may conduct multi-factor analyses to develop more comprehensive population dynamic models. Third, genetic structure was not investigated, so genetic exchange and inbreeding risks remain unclear. Further genetic monitoring using molecular tools will provide a basis for population genetic management [75]. Fourth, the annual population estimates represent maximum direct counts without formal measures of counting uncertainty; while the open wetland habitat and the dry-season concentration of deer limit counting error, such uncertainty may become increasingly relevant as the population grows, and future surveys should incorporate methods such as distance sampling or mark–resight analysis to quantify it.

## 5. Conclusions

The Milu deer in Dongting Lake is the world’s only naturally rewilded population of this species. Its continuous monitoring data from 2006 to 2025 provide a valuable sample for exploring the population dynamic rules of natural recovery of endangered wetland ungulates. This study is based on synchronous survey data of Milu deer in Dongting Lake from 2006 to 2025, adopting exponential and logistic growth models. It performs parameter estimation by combining Bayesian inference and NLS, constructs exponential and logistic growth models for the Milu deer population in Dongting Lake, systematically analyzes the population’s dynamic characteristics, simulates the effects of population supplementation, and quantifies the driving effects of hydrological factors. The Dongting Lake Milu deer population is the only fully wild population globally that originates from natural dispersal and has not undergone artificial wildness adaptation training. It originated from individuals that dispersed from the SMN after the 1998 flood, and subsequently achieved genetic admixture via two population supplementation events in 2016 and 2020, forming unique population characteristics distinct from populations under excessive human intervention, thereby providing an irreplaceable paradigm for research on the wild recovery of endangered species.

1. Population Characteristics and Trends

From 2006 to 2025, the Milu deer population in Dongting Lake grew from 27 individuals to 400 individuals, with an average annual growth rate of 0.16 ± 0.03, and the fitted models indicate that rapid population growth is likely to continue in the near term, followed by a gradual slowdown as the population approaches the estimated carrying capacity. Both the exponential and logistic models show extremely high goodness-of-fit (R^2^ ≥ 0.98), with intrinsic growth rates (*r*) of 0.13 and 0.16, respectively. The environmental carrying capacity (*K*) estimated by Bayesian inference is 905.1 (95% CI: 730–997), and the inflection point of population growth is projected to occur at year 22–23 (corresponding to 452.6 individuals), indicating that the population will soon enter the deceleration phase of logistic growth and approach the upper limit of environmental carrying capacity in about 50 years.

2. Effects of Population Supplementation Measures

The population supplementation measures involving the release of 26 Milu deer in 2016 and 2020 have a significant positive effect on population recovery. Simulations show that compared with the non-supplementation scenario, the intrinsic growth rates of the exponential and logistic models increase by 8.33% and 14.29%, respectively; from 2016 to 2025, the average annual population increment under the supplementation scenario reaches 28.70 ± 7.64 individuals, which is 14.80% higher than that of the non-supplementation scenario. This suggests that moderate population supplementation may help expand the population base, potentially alleviate inbreeding depression, and enhance the momentum of population growth.

3. Factors Affecting Population Dynamics

Hydrological fluctuations during flood season constitute the primary abiotic factor limiting Milu deer population growth. These adverse effects can be effectively mitigated through targeted conservation measures, including population supplementation and habitat improvement (e.g., restoring lake floodplains above flood level as flood refuges for Milu deer). Before the operation of Milu deer refuge platforms (2016–2023), population size showed a significant negative correlation with the maximum surface water area during the flood season (*r* = −0.89, *n* = 8, *p* <0.01); after the refuge platforms were put into use in 2024, the synergistic effect of population supplementation and habitat improvement offset hydrological stress, making the correlation insignificant across the almost full study period (2006–2024). This result verifies the important value of the coordinated implementation of multiple conservation measures in mitigating natural stress, although the evidence remains correlational and the post-implementation period of the refuge platforms is short.

4. Applicability and Effectiveness of Parameter Estimation Methods

The combination of Bayesian inference and NLS provides a cross-check of the parameter estimates obtained from the same observed time series. The intrinsic growth rates (*r* = 0.156) estimated by the two methods are consistent, and the intercept parameters (*a*) are highly coincident; Bayesian inference additionally quantifies parameter uncertainty through its posterior distributions and shows a slightly higher fitting accuracy in this study (RMSE = 13.59). However, this agreement should not be regarded as independent validation, because both methods are fitted to the same dataset and the population has not yet approached the saturation plateau of the logistic curve.

In summary, driven by the dual effects of natural adaptation and targeted conservation measures, the Milu deer population in Dongting Lake has achieved successful reestablishment from accidental natural dispersal to stable reproduction, providing a useful practical model for the wild recovery of endangered species. The core enlightenment of this case is that endangered species conservation should adhere to the principle of “nature-oriented, human-supported”: fully respecting the natural adaptive potential of species, prioritizing habitat integrity and connectivity, and implementing targeted conservation measures (such as facilitating natural dispersal, autonomous rewilding, habitat improvement, and population supplementation) based on population dynamic monitoring, so as to avoid excessive artificial disturbance. The findings of this study not only provide a scientific basis for the long-term conservation, habitat management, and genetic planning of Milu deer in Dongting Lake, but also offer important case references and methodological support for the reintroduction and wild recovery of other endangered large wetland ungulates affected by hydrological fluctuations. This study reflects the practical effectiveness of regional endangered species conservation and provides a concrete example for the application of nature-based solutions in biodiversity conservation.

## Figures and Tables

**Table 1:** Curve fitting results of population dynamics with different models.

Model	R^2^	F	df1	df2	Sig.	Remark
Linear	0.91	174.49	1	18	0.000	
Quadratic	0.98	465.64	2	17	0.000	
Cubic	0.98	408.10	3	16	0.000	
Power	0.90	156.54	1	18	0.000	
S	0.57	23.44	1	18	0.000	
Growth	0.98	912.18	1	18	0.000	Highest R^2^ and F
Exponential	0.98	912.18	1	18	0.000	Highest R^2^ and F
Logistic	0.98	912.18	1	18	0.000	Highest R^2^ and F

Note: R^2^, coefficient of determination; F, ANOVA F value; df1, numerator degrees of freedom; df2, denominator degrees of freedom; Sig., significance.

**Table 2:** Sensitivity analysis of annual reproductive output (*N*) to the female reproductive participation rate (*p*) and fawn survival rate (*s*) in the population supplementation scenario.

Parameter	Value	Δ*x*/*x*_0_	*N*	Δ*N*/*N*_0_	*S*	Remark
*p*	0.50	−0.25	5.85	−0.25	1	-
*p*	0.60	−0.10	7.02	−0.10	1	-
*p*	0.67	0.00	7.839	0.00	-	Baseline
*p*	0.70	0.04	8.19	0.04	1	-
*p*	0.80	0.19	9.36	0.19	1	-
*s*	0.60	−0.23	6.03	−0.23	1	-
*s*	0.70	−0.10	7.035	−0.10	1	-
*s*	0.78	0.00	7.839	0.00	-	Baselane
*s*	0.80	0.03	8.04	0.03	1	-
*s*	0.90	0.15	9.045	0.15	1	-

Notes: *p* is the female reproductive participation rate; *s* is the fawn survival rate; *x*_0_ and *N*_0_ are the baseline values of parameter *x* and annual reproductive output *N*; Δ*x*/*x*_0_ and Δ*N*/*N*_0_ are their relative changes; *S* is the sensitivity index. *F* is the total number of supplemented females = 15; *N* = *F* × *p* × *s*, hence *S* is constant at 1 (proportional response); *p*_0_ = 0.67 and *s*_0_ = 0.78 are the baselines, giving *N*_0_ = 7.839.

**Table 3:** Annual population increase and growth rate of Milu deer under population supplementation and no-supplementation scenarios simulated in Dongting Lake (2006–2025).

Year Period	Population with Supplementation		Population Without Supplementation	
	Annual Increase	Annual Growth Rate	Annual Increase	Annual Growth Rate
2006–2015	9.55 ± 2.65	0.19 ± 0.06	9.55 ± 2.65	0.19 ± 0.06
2016–2025	28.70 ± 7.64	0.14 ± 0.03	25 ± 8.7	0.13 ± 0.04
2006–2025	19.63 ± 4.58	0.16 ± 0.03	18 ± 4.87	0.16 ± 0.04

**Table 4:** Comparison and analysis of intrinsic growth rates for population growth models of Milu deer in the supplementation and non-supplementation scenarios.

Intrinsic Growth Rate	Supplementation	No-Supplementation	Increase (%)
*r* value of exponential model	0.13	0.12	8.33
*r* value of logistic model	0.16	0.14	14.29

**Table 5:** Pearson correlation coefficients between Milu deer population size and maximum water surface area across different study periods under varying targeted conservation measures.

Period	*r*	*n*	*p*-Value	Sig.	Remarks
2006–2015	−0.36	10	0.307	ns	No SUP; no RP
2006–2019	−0.03	14	0.919	ns	First SUP (2016); no RP
2016–2019	−0.69	4	0.310	ns	First SUP before second SUP; no RP
2016–2023	−0.89	8	0.003	**	Second SUP (2020); no RP; 2016–2020 subset
2016–2024	−0.18	9	0.643	ns	Second SUP (2020); RP activated; 2016–2020 subset
2006–2023	−0.39	18	0.110	ns	Second SUP (2020); no RP
2019–2023	−0.91	5	0.032	*	Before second SUP and no RP
2006–2024	−0.10	19	0.684	ns	Two SUP events; RP activated; extreme flood year (2024)
2006–2025	−0.08	20	0.737	ns	Two SUP events; RP activated

Sig., significance level; ns, not significant; *, significant at *p* < 0.05; **, significant at *p* < 0.01; SUP, supplementation; RP, refuge platform.

## Data Availability

The data that support the findings of this study are available on request from the corresponding author.

## References

[1] Hoffmann M., Duckworth J.W., Holmes K., Mallon D.P., Rodrigues A.S.L., Stuart S.N. (2015). The Difference Conservation Makes to Extinction Risk of the World’s Ungulates. Conserv. Biol..

[2] Senior R.A., Bagwyn R., Leng D., Killion A.K., Jetz W., Wilcove D.S. (2024). Global Shortfalls in Documented Actions to Conserve Biodiversity. Nature.

[3] IUCN/SSC (2013). Guidelines for Reintroductions and Other Conservation Translocations.

[4] Carver S., Convery I., Hawkins S., Hertel S., Fallon J., Lyons K., Beyers R., Locquet A., Derham T., Kun Z. (2025). Guidelines for Rewilding.

[5] Bakker E.S., Svenning J.-C. (2018). Trophic Rewilding: Impact on Ecosystems under Global Change. Philos. Trans. R. Soc. B Biol. Sci..

[6] Carver S., Hawkins S., Convery I., Beyers R., Derham T., Cao Y. (2025). Rewilding: Ten Years of Evolution and Development. Annu. Rev. Environ. Resour..

[7] Thompson P.D., Lundskog C.L., Dittmer D.E. (2022). A Successful Reintroduction of Columbia Spotted Frog (*Rana luteiventris*) through Repatriation of Recently Hatched Larvae. J. Herpetol..

[8] Temsiripong Y., Woodward A.R., Ross J.P., Kubilis P.S., Percival H.F. (2006). Survival and Growth of American Alligator (*Alligator mississippiensis*) Hatchlings after Artificial Incubation and Repatriation. J. Herpetol..

[9] Kaczensky P., Ganbaatar O., von Wehrden H., Enksaikhan N., Lkhagvasurren D., Walzer C. (2007). Przewalski’s Horse (*Equus Ferus Przewalskii*) Re-Introduction in the Great Gobi B Strictly Protected Area: From Species to Ecosystem Conservation. Mong. J. Biol. Sci..

[10] Böhm C., Bowden C.G.R., Seddon P.J., Hatipoğlu T., Oubrou W., El Bekkay M., Quevedo M.A., Fritz J., Yeniyurt C., Lopez J.M. (2021). The Northern Bald Ibis Geronticus Eremita: History, Current Status and Future Perspectives. Oryx.

[11] Harding G., Griffiths R.A., Black S.A. (2025). Factors Influencing Species Reintroduction Success. Biodivers. Conserv..

[12] Xue D.Y., Zhang Y.Y., Cheng Z.B., Zhong Z.Y., Cao M., Fu M.D., Bai J.D., Yuan X.J. (2022). Père David’s Deer (*Elaphurus davidianus*) in China: Population Dynamics and Challenges. J. Resour. Ecol..

[13] Pei P., Wang L., Shao Y., Shi C., Bao X. (2018). Reintroduced Przewalski’s Horses: Breeding Success and Population Viability Analysis in Anxi National Nature Reserve. Acta Theriol. Sin..

[14] Yang Z.S., Gu X.D., Nie Y.G., Huang F., Huang Y., Dai Q., Hu Y., Yang Y., Zhou X., Zhang H. (2018). Reintroduction of the Giant Panda into the Wild: A Good Start Suggests a Bright Future. Biol. Conserv..

[15] Chen W.G., Li X., Liu C., Wang L., Yu X.P. (2013). Status of a Reintroduced Population of the Crested Ibis (*Nipponia nippon*) in Ningshan County Shaanxi Province. Chin. J. Wildl..

[16] Wang Z.H., Yao H., Ding Y.Z., Thorbjarnarson J., Wang X.M. (2011). Testing Reintroduction as a Conservation Strategy for the Critically Endangered Chinese Alligator: Movements and Home Range of Released Captive Individuals. Chin. Sci. Bull..

[17] Cheng Z., Tian X., Zhong Z., Li P., Sun D., Bai J., Meng Y., Zhang S., Zhang Y., Wang L. (2021). Reintroduction, Distribution, Population Dynamics and Conservation of a Species Formerly Extinct in the Wild: A Review of Thirty-Five Years of Successful Milu (*Elaphurus davidianus*) Reintroduction in China. Glob. Ecol. Conserv..

[18] Li P.F., Ding Y.H., Zhang Y.M., Yang T., Song Y.C., Cai J.Q., Yao Y. (2018). Distribution and Population Investigation of Wild Père David’s Deer in the Middle Reaches of Yangtze River. Chin. J. Wildl..

[19] Li C.L., Tian S.R., Song Y.C., Zhang H., Gao D.L., Yang D.D., Gui X.J. (2023). Population Viability Analysis of Père David’s Deer (*Elaphurus davidianus*) in Dongting Lake, Hunan Province. Acta Theriol. Sin..

[20] Yang D.D., Jiang Z.G., Ma J.Z., Hu H.J., Li P.F. (2005). Causes of endangerment or extinction of some mammals and its relevance to the reintroduction of Père David’s deer in the Dongting Lake drainage area. Biodivers. Sci..

[21] Cheng Z., Zhang Y., Liu W., Zhong Z., Bai J., Ma J., Guo Q., Li P., Zhang H., Xiong B. (2025). Population Dynamics and the Role of Protected Areas in China’s Milu Deer (*Elaphurus davidianus*) Rewilding. Sci. Rep..

[22] Clark-Wolf T.J., St. John J., Rajesh C.A., Hebblewhite M. (2025). Caribou and Reindeer Population Cycles Are Driven by Top-Down and Bottom-Up Mechanisms Across Space and Time. Ecol. Evol..

[23] Jiang Q., Xiao H., Zhou H., Li Y., Fu J., Meshach A., Li Q., Kang L., Yan L., Shu Y. (2025). Spatiotemporal Niche Differentiation of Ungulates in the Southwest Mountains, China. Animals.

[24] Angert A.L., Crozier L.G., Rissler L.J., Gilman S.E., Tewksbury J.J., Chunco A.J. (2011). Do Species’ Traits Predict Recent Shifts at Expanding Range Edges?. Ecol. Lett..

[25] Chen I.-C., Hill J.K., Ohlemüller R., Roy D.B., Thomas C.D. (2011). Rapid Range Shifts of Species Associated with High Levels of Climate Warming. Science.

[26] Gaillard J.-M., Festa-Bianchet M., Yoccoz N.G., Loison A., Toigo C. (2000). Temporal Variation in Fitness Components and Population Dynamics of Large Herbivores. Annu. Rev. Ecol. Syst..

[27] Ma J., Bai J., Su R., Ma H., Feng C., Zhong Z., Guo Q., Zhang Q., Cheng Z., Cheng K. (2025). Behavioral Plasticity of Rewilding Milu in Mountainous Region of Northern China. Animals.

[28] Jiang Z., Yu C., Feng Z., Zhang L., Xia J., Ding Y., Lindsay N. (2000). Reintroduction and Recovery of Père David’s Deer in China. Wildl. Soc. Bull..

[29] Song Y.C., Yang D.D., Zou S.J., Li P.F., Zhang H., Wen H.J., Jiang Z.G. (2015). Sex-biased dispersal in naturally re-wild Milu in the Dongting Lake Region, China. Acta Ecol. Sin..

[30] Wang S.Y., Zhao Y.L., Xu Z.G., Li L., Wu L., Duan C.C., Peng J. (2017). Behavioural Rhythms during the Adaptive Phase of Introduced Milu/Père David’s Deer (*Elaphurus davidianus*) in the Dongting Lake Wetland, China. Pak. J. Zool..

[31] Yang D.D., Jiang Z.G., Cao T.R., Wen S.Z., Zhao K.J., Gui X.J., Xu Y.X. (2002). Feasibility of reintroducing Père David’s deer (*Elaphurus davidianus*) to the Dongting Lake region, Hunan Province. Biodivers. Sci..

[32] Lai X.J., Jiang J.H., Huang Q. (2013). Effects of the Normal Operation of the Three Gorges Reservoir on Wetland Inundation in Dongting Lake, China: A Modelling Study. Hydrol. Sci. J..

[33] Li Y.Z., Bian H.L., Ren B., Xie Y.H., Ding X.H., Yao X., Zhou Q.Q. (2019). Morphological Responses of Two Plant Species from Different Elevations in the Dongting Lake Wetlands, China, to Variation in Water Levels. Nord. J. Bot..

[34] Tian W.G., Peng J.D., Shen J., Li S. (2012). Sequence analysis on the area of Dongting Lake before and after building three gorges dam based on MODIS image sequences. J. Anhui Agric. Sci..

[35] Guan L., Lei J.L., Zuo A.J., Zhang H., Lei G.C., Wen L. (2016). Optimizing the Timing of Water Level Recession for Conservation of Wintering Geese in Dongting Lake, China. Ecol. Eng..

[36] Cao L., Barter M., Lei C. (2008). New Anatidae Population Estimates for Eastern China: Implications for Current Flyway Estimates. Biol. Conserv..

[37] Zheng P., Guo C., Dong G., Zhou X., Tang S., Mi M., Dai J., Jiang T., Zeng Q., Liu H. (2026). Research and Practice on Integrated Ecological Protection and Restoration of Mountains, Rivers, Forests, Farmlands, Lakes, Grasslands and Deserts in the Dongting Lake Area. Acta Ecol. Sin..

[38] Peng Y., He G., Wang G., Cao H. (2021). Surface Water Changes in Dongting Lake from 1975 to 2019 Based on Multisource Remote-Sensing Images. Remote Sens..

[39] Zhang Y., Jiang C., Long Y., Yan S. (2024). Dynamic Monitoring of Water Area in Dongting Lake during Flood Season based on Sentinel-1A SAR Data. Remote Sens. Technol. Appl..

[40] Ministry of Water Resources of the People’s Republic of China (2022). China Hydrological Yearbook 2021.

[41] Ministry of Water Resources of the People’s Republic of China (2023). China Hydrological Yearbook 2022.

[42] Ministry of Water Resources of the People’s Republic of China (2024). China Hydrological Yearbook 2023.

[43] Ministry of Water Resources of the People’s Republic of China (2025). China Hydrological Yearbook 2024.

[44] Ministry of Water Resources of the People’s Republic of China (2026). China Hydrological Yearbook 2025.

[45] R Core Team (2013). R: A Language and Environment for Statistical Computing.

[46] Heydari J., Lawless C., Lydall D.A., Wilkinson D.J. (2014). Fast Bayesian Parameter Estimation for Stochastic Logistic Growth Models. Biosystems.

[47] Iijima H., Nagaike T., Honda T. (2013). Estimation of Deer Population Dynamics Using a Bayesian State-Space Model with Multiple Abundance Indices. J. Wildl. Manag..

[48] Bates D.M., Watts D.G. (1988). Nonlinear Regression Analysis and Its Applications.

[49] Bergqvist G., Kindberg J., Elmhagen B. (2024). From Virtually Extinct to Superabundant in 35 Years: Establishment, Population Growth and Shifts in Management Focus of the Swedish Wild Boar (*Sus scrofa*) Population. BMC Zool..

[50] Reyns N., Casaer J., Smet L.D., Devos K., Huysentruyt F., Robertson P., Verbeke T., Adriaens T. (2018). Cost-Benefit Analysis for Invasive Species Control: The Case of Greater Canada Goose Branta Canadensis in Flanders (Northern Belgium). PeerJ.

[51] Montgomery D.C., Peck E.A., Vining G.G. (2012). Introduction to Linear Regression Analysis.

[52] de Kroon H., Plaisier A., van Groenendael J., Caswell H. (1986). Elasticity: The Relative Contribution of Demographic Parameters to Population Growth Rate. Ecology.

[53] Shapiro S.S., Wilk M.B. (1965). An Analysis of Variance Test for Normality (Complete Samples). Biometrika.

[54] Bishara A.J., Hittner J.B. (2012). Testing the Significance of a Correlation with Nonnormal Data: Comparison of Pearson, Spearman, Transformation, and Resampling Approaches. Psychol. Methods.

[55] Cohen J. (1988). Statistical Power Analysis for the Behavioral Sciences.

[56] Tsoularis A., Wallace J. (2002). Analysis of Logistic Growth Models. Math. Biosci..

[57] Li C.X., Jiang L.N., Shao Y., Zhang D.J. (2013). Biostatistics.

[58] Sun L.Y. (1985). Scientific Management and Mathematical Models of Population.

[59] Sun R.Y., Wang D.H., Niu C.J., Liu D.Z., Zhang L. (2019). Principles of Animal Ecology.

[60] Yang L.W. (2023). Prediction of Habitat Suitability and Population Viability Analysis for Père David’s Deer (*Elaphurus davidianus*). Master’s Thesis.

[61] Huang J.B., Zhang G.H. (1990). Comparison of Theory and Case Fitting between Logistic and Cui-Lawson Population Increment Models. Chin. J. Appl. Ecol..

[62] Seddon N., Smith A., Smith P., Key I., Chausson A. (2021). Getting the Message Right on Nature-Based Solutions to Climate Change. Glob. Change Biol..

[63] Seddon N., Chausson A., Berry P., Girardin C.A.J., Smith A., Turner B. (2020). Understanding the Value and Limits of Nature-Based Solutions to Climate Change and Other Global Challenges. Philos. Trans. R. Soc. B Biol. Sci..

[64] Yue Y., Wang Y., Ye Z., Zhang C., Qiu L., Xu Q., He X., Yuan Y., Yang X., Dong S. (2024). Ecological and Public Advantages of a Dual Flagship Strategy: Giant Panda and Snow Leopard. Diversity.

[65] Yang J.J., Dai Z.J., Mei X.F., Bu F.Y., Liu Y.Z. (2026). Hydrological Alterations Induced Lakeward Expansion of Wetland Vegetation in Dongting Lake, China’s Second-Largest Lake. CATENA.

[66] Yang D.D., Song Y.C., Ma J.Z., Li P.F., Zhang H., Price M.R.S., Li C.L., Jiang Z.G. (2016). Stepping-Stones and Dispersal Flow: Establishment of a Meta-Population of Milu (*Elaphurus davidianus*) through Natural Re-Wilding. Sci. Rep..

[67] MacArthur R.H., Wilson E.O. (1967). The Theory of Island Biogeography.

[68] Hanski I., Ovaskainen O. (2000). The Metapopulation Capacity of a Fragmented Landscape. Nature.

[69] Bar-David S., Saltz D., Dayan T., Perelberg A., Dolev A. (2005). Demographic Models and Reality in Reintroductions: Persian Fallow Deer in Israel. Conserv. Biol..

[70] Leberg P.L. (1993). Strategies for Population Reintroduction: Effects of Genetic Variability on Population Growth and Size. Conserv. Biol..

[71] Bowler D.E., Nilsen E.B., Bischof R., O’Hara R.B., Yu T.T., Lynam C.P. (2019). Integrating Data from Different Survey Types for Population Monitoring of an Endangered Species: The Case of the Eld’s Deer. Sci. Rep..

[72] Beissinger S.R., Westphal M.I. (1998). On the Use of Demographic Models of Population Viability in Endangered Species Management. J. Wildl. Manag..

[73] Mo X.Y., Lei Y.W., Cui X.Y., Mao X.K., Li H.H., Chen J., Kang Z.J., He C.R., Liu Z.X., Zhang H. (2025). Systematic Description of Cetartiodactyla in Hunan Province Based on Museum Specimens and Historical Literature. Life Sci. Res..

[74] Li Y., Shan J., Li J., Yuan F., Miao L., Xie G. (2013). Research Progress on Ecology of Chinese Water Deer (*Hydropotes inermis*). Chin. Wildl..

[75] Zhang S., Li C., Li Y., Chen Q., Hu D., Cheng Z., Wang X., Shan Y., Bai J., Liu G. (2021). Genetic Differentiation of Reintroduced Père David’s Deer (*Elaphurus davidianus*) Based on Population Genomics Analysis. Front. Genet..

